# 

**Published:** 1886-07

**Authors:** 


### CONTENTS.

COMMUNICATIONS.

Every Day Practice ........................................ 321–325
The Treatment of Pulpless and Gaseous Teeth.... .................. 326
Address ........................................ 328

PROCEEDINGS.

Alabama State Dental Association ........................................ 337

EDUCATIONAL.

Graduates in Dental Surgery for the Year 1886 .................. 356

SALMAGUNDI ........................................ 364

SOCIETY MEETINGS ........................................ 366

EDITORIAL.

Distinguished Honor ........................................ 370

OBITUARY.

In Memory of Dr. W. Hart Cameron ........................................ 372

